# Preliminary study on the mechanism of OPN up-regulating PD-L1 to induce CD8 + T cell exhaustion in synovial sarcoma

**DOI:** 10.1186/s12885-026-16363-x

**Published:** 2026-07-03

**Authors:** Huifang Zhuo, Xinyi Li, Zhongxu Xue, Wenyu An, Lian Meng, Wei Jia, Ning Wang

**Affiliations:** 1 Department of Pathology, Department of Pathology, the First Affiliated Hospital, Shihezi University School of Medicine, Shihezi University, Xinjiang, 832002 China; 2 The pathological diagnosis Clinical Medical Research Center of Xinjiang Production and Construction Corps, Shihezi, 832061 China; 3 Guangming Traditional Chinese Medicine Hospital of Pudong New Area, Shanghai, 201399 PR China

**Keywords:** Synovial sarcoma, OPN, PD-L1, CD8 + T cells, Escape of immunity

## Abstract

**Background:**

Synovial sarcoma (SS) is a rare and highly malignant soft tissue sarcoma. The efficacy of existing therapies is limited, and new strategies are urgently needed. Osteopontin (OPN) promotes disease progression by regulating tumor-immune cell interaction and immunosuppressive microenvironment, which is a potential therapeutic target. The aim of this study was to investigate the role of OPN in SS immune regulation and tumor progression.

**Methods:**

A stable cell line with OPN knockdown was constructed using lentiviral transduction. RNA-seq and bioinformatics analysis were used to predict the correlation between OPN and immunity. Flow cytometry and ELISA were used to detect the changes in the number and function of CD8 + T cells. EDU, CCK-8 and Transwell chamber experiments were used to detect the proliferation, invasion and migration ability of SS. The protein interaction between OPN and PD-L1 was detected by molecular docking and Co-IP. The expression of PD-L1 was detected by Western blot and flow cytometry. The expression levels of OPN, PD-L1 and CD8 were detected by immunohistochemistry. To predict the correlation of OPN, PD-L1 and CD8 in sarcoma tissues and their clinical prognostic significance.

**Results:**

RNA-seq analysis revealed that OPN-associated differentially expressed genes were significantly enriched in immune response–related biological processes. Bioinformatics analysis demonstrated that OPN expression in sarcoma was associated with immune responses and T-cell activation, and was negatively correlated with immune cell infiltration. OPN induced CD8⁺ T-cell exhaustion, thereby promoting immune evasion and facilitating the proliferation, invasion, and migration of synovial sarcoma (SS) cells. Molecular docking and co-immunoprecipitation assays confirmed a direct protein–protein interaction between OPN and PD-L1, and knockdown of OPN resulted in a significant reduction in PD-L1 expression. In SS tissues, OPN was highly expressed, whereas PD-L1 and CD8 were expressed at low levels and exhibited a positive correlation. Furthermore, bioinformatics analysis showed that PD-L1 expression was positively correlated with CD8 expression in sarcoma, and low CD8 expression was associated with poor prognosis.

**Conclusions:**

We preliminarily propose that OPN promotes immune evasion in synovial sarcoma (SS) by regulating PD-L1 expression and subsequently inducing CD8⁺ T-cell exhaustion. This mechanism suggests that OPN may serve as a novel immunotherapeutic target and provides new insights into the development of immunotherapy strategies for SS.

**Supplementary Information:**

The online version contains supplementary material available at https://doi.org/10.1186/s12885-026-16363-x.

## Background

Synovial Sarcoma (SS) is a rare and highly malignant Soft Tissue Sarcoma, accounting for about 5–10% of all soft tissue sarcomas (STS), and it often occurs in young adults [1]. The molecular pathological features of SS are t (X; 18) (p11; q11) chromosomal translocation leads to the formation of SS18-SSX fusion gene, which is considered to be the main driving factor for the occurrence and development of SS [2]. SS mainly occurs in the limbs, but can also occur in other parts of the body, including trunk, head and neck, abdomen, etc [3, 4]. In 2020, the World Health Organization (WHO) defined SS as a mesenchymal spindle cell tumor [5, 6], characterized by biphasic differentiation of epithelial and spindle tumor cells, and histologically divided into the following three types based on the different proportions of epithelioid and spindle cell composition: Spindle cell synovial sarcoma (i.e., monophasic fibrous synovial sarcoma, Monophasic Fibrous Synovial Sarcoma (MFSS), Biphasic Synovial Sarcoma, monophasic fibrous synovial sarcoma (MFSS) BSS) and Poorly Differentiated Synovial Sarcoma (PDSS) [7]. The clinical prognosis of SS is poor, with a high risk of late recurrence and metastasis (mainly to the lungs or pleura), and since it usually occurs after 5 years, at least 10 years of follow-up is recommended [8–10]. At present, the standard treatment of SS mainly includes surgical resection, combined with chemotherapy and radiotherapy [11, 12], but there are few effective treatment options for patients with refractory or recurrent/metastatic SS [9]. Therefore, the limitations in the treatment of SS make the development of new therapies, especially for the treatment of metastatic cases, an urgent research need.

In recent years, molecular targeted therapy and cellular immunotherapy have attracted much attention as new cancer treatment methods. In the direction of molecular targeted therapy for SS, a variety of new drugs have been studied in clinical trials, but so far only pazoparib (a receptor tyrosine kinase inhibitor) has been clinically approved [13]. The application of cellular immunotherapy in SS is still in the exploratory stage, and the current research mainly focuses on Immune Checkpoint Inhibitors (ICIs), adoptive T cell therapy and tumor vaccines [14]. Among them, ICIs have played an important role in tumor immunotherapy, especially in the clinical treatment of a variety of malignant tumors, such as melanoma [15], lung cancer [16], renal cell carcinoma [17], etc. However, not all cancers respond to ICIs immunotherapy, and the differences in treatment response may be related to multiple factors such as the tumor immune microenvironment, mutation burden, expression level of immune checkpoint molecules, and individual differences in patients. Programmed death-ligand 1 (PD-L1) and Programmed Cell Death Protein 1 (PD-1) have been confirmed to be important immune checkpoints for tumor immune escape [18]. The latest study by Mai He et al. found that SS has a low tumor mutation load and a low expression level of PD-L1 [19], indicating that the immunogenicity of SS is weak, and the immune system is difficult to effectively identify and attack SS cells, so that the therapeutic effect of ICIs in SS is limited. In order to improve the therapeutic effect of SS, there is an urgent need to explore new therapeutic targets and strategies to enhance the ability of the immune system to recognize and kill tumors.

In our previous study, we identified 336 differentially expressed genes (DEGs) using laser capture microdissection (LCM) combined with exon array technology. Among them, extracellular matrix (ECM)-related genes, predominantly osteopontin (OPN), were significantly enriched in key signaling pathways of SS, suggesting their crucial roles in the pathogenesis and progression of SS [20]. OPN is also known as Secreted Phosphoprotein 1 (SPP1). In physiological state, OPN maintains the dynamic balance of tissue microenvironment by regulating angiogenesis, maintaining bone metabolism balance and immune homeostasis. However, under pathological conditions, OPN participates in the occurrence and development of diseases through multiple mechanisms such as promoting tumor progression, mediating distant metastasis, assisting immune escape, inducing angiogenesis, and enhancing chemotherapy resistance [21, 22]. Studies have shown that OPN is highly expressed in a variety of tumor types, including skin cancer, thyroid cancer, non-small cell lung cancer, hepatocellular carcinoma, pancreatic cancer, melanoma and osteosarcoma [23–26]. Our previous study confirmed that OPN was highly expressed in SS tissues and promoted the invasion and migration of SS cells through EMT through immunohistochemistry and cell functional experiments.

OPN is not only highly expressed in tumors, but also expressed in a variety of immune cells, including B cells, natural killer cells, macrophages, dendritic cells, monocytes and neutrophils [27–29]. Related experiments have shown that OPN plays a role in the tumor microenvironment by regulating macrophages and T cells [30–32]. The interaction between OPN+ macrophages and Cancer-Associated Fibroblasts (CAFS) can form a tumor immune barrier that affects the effect of immunotherapy. The loss of OPN would eliminate the inhibitory effect of CAFs on the immune microenvironment, thereby allowing killer T cells to infiltrate the tumor core and enhancing the sensitivity of tumor immune checkpoint inhibitors. In hepatocellular carcinoma, OPN promotes PD-L1 expression by activating the colony stimulating factor-1 (CSF-1)/CSF-1 receptor (CSF-1R) pathway in macrophages, and blocking PD-L1 and CSF1R can lead to increased CD8 + T cell infiltration [33]. Another study found that PD-L1 expression was down-regulated in macrophages lacking OPN, which further supports the regulatory role of OPN in PD-L1 expression [34]. In addition, OPN derived from tumor associated macrophages can up-regulate the expression of PD-L1 on the surface of non-small cell lung cancer cells through the NF-κB signaling pathway, thereby inhibiting the anti-tumor immune response [35].

In summary, this study aims to investigate the molecular mechanisms by which OPN regulates PD-L1 expression in synovial sarcoma (SS) cell lines and to elucidate its effects on immune cells, particularly CD8⁺ T cells, thereby uncovering the role of OPN in immune evasion in SS. These findings may provide a theoretical basis for the development of novel immunotherapeutic strategies for SS. By targeting OPN or its downstream signaling pathways, we may enhance the sensitivity of SS to immunotherapy, ultimately improving clinical outcomes for patients. Moreover, this study may offer new insights and valuable references for immunotherapy in other low-immunogenic tumors.

## Materials and methods

### Cell culture

SW982 human synovial sarcoma cell line derived from ATCC library was purchased from Shanghai Fuxiang Biological Co., LTD. The HS-SY-II cell line was gifted by Mr. Peng Changliang of the Second Affiliated Hospital of Shandong University School of Medicine. SW982 cells were cultured in DMEM medium containing 10% FBS and 1% penicillin and streptomycin, and HS-SY-II were cocultured in DMEM high glucose medium containing 10% FBS plus 1% double antibody. OPN knockdown and overexpression lentiviral vectors were commissioned to construct and synthesize Shanghai Jikai Gene. OPN knockdown cells (sh-NC/sh-OPN) were cultured in DMEM medium containing 10% fetal bovine serum, 1% penicillin and streptomycin, and 0.5ug/ml puromycin. OPN overexpression cells (OE-NC/OE-OPN) were cultured in DMEM medium containing 10% fetal bovine serum, 1% penicillin and streptomycin, and 0.25ug/ml puromycin. All cells were incubated in a constant temperature incubator (ESCO (Lexicon H) Technology Company) at 37 °C with 5% CO2.

### Tissue sample collection

A total of 31 cases of SS were collected from the Department of Pathology, the First Affiliated Hospital of Shihezi University from 1975 to 2022 (Table 1 in the Supplementary Material). After dehydration and fixation steps, all specimens were paraffin-embedded, sectioned, and stained with hematoxylin-eosin (HE) staining, and standard immunohistochemical detection was performed. Two senior pathologists participated in the reading and diagnosis of the slides. The TNM staging of each case strictly followed the latest staging system issued by the American Joint Committee on Cancer (AJCC). Histological grading was based on the standardized grading scheme established by the French Federation of Cancer Centres (FNCLCC).

**Table 1:** The expression levels and percentages of OPN, PD-L1, and CD8 in SS tissues

Variable	Number of Cases (*n*)	Percentage (%)
OPN Expression		
High Expression	17	54.8
Low Expression	14	45.2
PD-L1 Expression		
High Expression	8	25.8
Low Expression	23	74.2
CD8 Expression		
High Expression	10	32.3
Low Expression	21	67.7

### A stable cell line was constructed

OPN interference lentiviral vector (target sequence: CTTCAGGGTTATGTCTATGTT) was commissioned to construct and synthesize Shanghai Jikai Gene. Forty-eight hours after infection, the cells were cultured in medium containing 0.5 µg/ml puromycin (45 ml DMEM+5 ml serum+500 µl double antibody+25 µl puromycin). All transfection protocols followed the manufacturer’s guidelines.

### RNA extraction and qRT-PCR

Total RNA was extracted from cells using Trizol reagent (Invitrogen, USA) according to the manufacturer’s instructions. The extracted RNA was reverse transcribed into cDNA using a reverse transcription kit (Thermo, USA). qRT-PCR experiments were performed on a 7500 Fast Real-Time PCR system using a kit (TaKaRa, Japan). Results are expressed as fold change relative to GAPDH((2−∆∆Ct). This study used primers from Shanghai sangon company:beta actin ( F :5 ‘GAGCGGGAAATCGTCCGTGACATT3’,R:5‘GATGGAGTTGAAGGTAGTTTCGTG3’); OPN F:5′AGTTTCGCAGACCTGACATCC3′, R:5′TTCCTGACTATCAATCACATCGG 3′).

### Western blot

Cells were lysed in RIPA buffer containing a mixture of protease and phosphatase inhibitors (Solarbio, Beijing), and the obtained lysates were then separated by SDS-PAGE and transferred to a 0.45µM PVDF membrane (Solarbio, Beijing). The membrane was blocked with 5%BSA (Biosharp, Beijing) and incubated with primary antibodies for 14 h overnight. After washing with TBST, the membranes were incubated with secondary antibodies for 2 h at room temperature and exposed after washing with TBST.

### CCK8 assay

Cells in sh-NC and sh-OPN groups were digested with trypsin, counted by centrifugation to achieve a cell density of about 5 × 104 cells /ml, and seeded in 96-well plates with a volume of 100 µl per well. The next day, 100ul of CCK8 dilution was added to each well and incubated in the incubator for 2 h. The OD values of each group were tested at the same time points on day 1, day 2, day 3, and day 4.

### EDU

The proliferation was further quantified using an EDU kit from Shanghai Biyuntian, and the procedure was performed according to the instructions. After EDU staining, DAPI staining solution (Shanghai Biyuntian Company) was added, and the cells were left in the dark at room temperature for 15 min, washed, and then detected under the fluorescence microscope.

### Transwell assay

Invasion and migration assays were performed using Transwell chambers (Corning, USA) with or without pre-coated Matrigel (Corning, USA). “Cells (up to 3.5 × 104/200 µl in serum-free medium) were seeded into the upper chamber, whereas the lower chamber contained complete medium supplemented with 20% FBS.” After incubation for 24–48 h, the cells were fixed with 4% paraformaldehyde, stained with 0.1% crystal violet, and photographed under an inverted microscope.

### Co‑immunoprecipitation (Co‑IP)

SW982 cells were lysed with RIPA buffer, and the resulting lysate was centrifuged to separate the supernatant, which was subsequently spun with OPN antibody for 2–4 h at 4 ° C shaking, and the mixture was then added to 40 µl of protein agarose beads A/G and incubated at 4 ° C shaking for 10–12 h. The next day, the complexes were centrifuged, washed five times, cell lysate and loading buffer (loding buffer: RIPA: PMSF = 25:100:1) were added, and boiled for 8 min. After centrifugation, the resulting supernatants were loaded onto SDS-PAGE gels, followed by electrophoresis, and analyzed using Western blotting as described previously.

### Immunohistochemistry

Paraffin embedded tissues were sectioned, deparaffinized in xylene and rehydrated. EDTA was used for antigen repair. Sections were then incubated in H2O2 in the dark for 10 min and incubated with anti-CD8 and anti-PD-L1 overnight at 4 ° C. The following day, after incubation with the secondary antibody for 30 min, staining was performed. Sections were then counterstained with hematoxylin, dehydrated, and fixed.

Scoring criteria:

1. OPN scoring: OPN expression was evaluated based on the percentage of positive cells and staining intensity.

① Staining intensity was scored as follows: no staining (0 points), yellow (1 point), yellow-brown (2 points), and brown (3 points).

② The percentage of positive cells was scored as: <5% (0 points), 6%–25% (1 point), 26%–50% (2 points), 51%–75% (3 points), and 76%–100% (4 points).

The final score was obtained by summing the two parameters and graded as follows:– (0–1 points), + (2–4 points), ++ (5–8 points), and +++ (9–12 points).

In this study, –/+ were classified as low expression, whereas ++/+++ were classified as high expression.

(2) PD-L1 scoring: Random high-power fields were selected for evaluation.

Negative (0 points); low positive expression (1 point, 1%–5%); high positive expression (2 points, ≥ 5%).

In this study, scores of 0–1 were defined as low expression, and a score of 2 was defined as high expression.

(3) CD8 scoring: Random high-power fields were selected for counting.

No positive cells (0 points); 1–10 positive cells (1 point); 11–50 positive cells (2 points); 51–100 positive cells (3 points); >100 positive cells (4 points).

In this study, scores of 0–1 were classified as low expression, whereas scores of 2–4 were classified as high expression.

### Tumor cells were co-cultured with human peripheral blood mononuclear cells (PBMC)

Peripheral venous blood was collected from healthy volunteers, transferred into a 15-mL centrifuge tube, and subsequently diluted with ice-cold PBS. The volume ratio of peripheral blood and PBS was 1:1. The human peripheral blood lymphocyte separation solution (Tianjin Haoyang Biological) was added into a new centrifuge tube, and the ratio of separation solution and diluted peripheral blood was 1:1. The cells in the middle membrane layer were diluted in PBS, mixed, centrifuged, and co-cultured for 48 h according to the ratio of tumor cells: PBMC = 1:2.

### Flow cytometry

Flow cytometry was used to detect changes in the number of cells expressing CD8 markers: After 48 h of co-culture, the medium containing PBMC was centrifuged, and 1 × 106 cells were collected and resuspended in 95 µl PBS, and 5µL CD3 and CD8 antibodies were added respectively to make the final reaction volume of 100µL. The cells were mixed and incubated at 4℃ in the dark for 30 min before detection. Analysis was performed using a flow cytometer (BIO-RAD, USA).

The expression of PD-L1 was detected by flow cytometry: 1 × 106 SS cells in sh-NC group and sh-OPN group were collected and resuspended by washing with 95ul PBS, and PD-L1 flow antibody was added to make the final volume of 100 µl. The cells were incubated in the dark at 4 degrees for 30 min before detection.

### ELISA

The expression levels of tumor necrosis factor-α (TNF-α), interferon-γ (IFN-γ) and granzyme B (Granzyme B) were detected by ELISA kit. After the cells of knockdown group were co-cultured with PBMC for 48 h, the supernatant after co-culture was collected, and blank Wells, standard Wells and sample Wells were set up. The experimental operation was carried out strictly according to the instructions of the kit. The OD values at 450 nm and 570 nm wavelengths were determined using a microplate reader.

### RNA sequencing

RNA was extracted from sh-NC and sh-OPN group cells, and the extracted RNA samples were sent to a professional institution (Shanghai Bayq Biomedical Technology Co., LTD., China) for quality detection and library construction. After the library inspection was qualified, lumina sequencing was performed.

#### Data analysis

Differential expression analysis: DESeq2 software (version 1.20.0) was used for differential expression analysis to compare the gene expression differences between sh-NC group and sh-OPN group. Genes with |log2FoldChange|>1 and corrected *p* value (padj) < 0.05 were defined as significantly differentially expressed genes (DEGs).

Functional enrichment analysis: clusterProfiler software (version 3.8.1) was used to perform GO functional enrichment analysis and KEGG pathway enrichment analysis of differentially expressed genes. Significantly enriched GO entries and KEGG pathways were defined as *p*-values < 0.05.

Gene set enrichment analysis (GSEA) : data sets were to GO and KEGG gene enrichment analysis, specific link to http://www.broadinstitute.org/gsea/index.jsp.

Protein-protein interaction (PPI) network analysis: The differentially expressed genes were constructed by STRING database, and the core node genes were screened and their functional modules were analyzed to reveal the potential molecular regulatory network.

### Reliability analysis

Analysis of OPN expression in sarcomas: Gene expression data of sarcoma patients (*n* = 265) in TCGA and gene expression data of skeletal muscle (*n* = 395) in GTEx were downloaded from the UCSC Xena (https://xenabrowser.net) website. The two groups of data were integrated to pick out the expression of OPN and imported into the wechat website (http://113.44.3.163/) for box plot.

The correlation between OPN and immune cells was analyzed. The data of sarcoma patients were collected from TCGA database, and the correlation between OPN and various immune cells was made on Gepia2 website (http://gepia2.cancer-pku.cn).

To analyze the survival prognosis of OPN, PD-L1 and CD8: in GEPIA2 (http://gepia2.cancer-pku.cn) website, select the markers of OPN, PD-L1 and CD8 to make the survival analysis in sarcoma, and select the Group Cutoff value: OPN: Cutoff-High :50%, Cutoff-Low:50%; PD-L1: Cutoff High :50%, Cutoff Low:50%; CD8: Cutoff High :50%, Cutoff Low:50%;

The correlation between OPN and PD-L1 and CD8 was analyzed. OPN, PD-L1 and CD8 genes were selected from GEPIA2 (http://gepia2.cancer-pku.cn) website for analysis.

To analyze the biological function of OPN: the data of sarcoma patients were collected from TCGA database, and the correlation between OPN and immune cell characteristic molecules was analyzed. The top 1000 genes with correlation were selected and imported into Sangerbox website (http://www.sangerbox.com), and GO analysis was performed.

### Molecular docking

OPN and PD-L1 protein sequences were obtained from UniProt database, and then modeled by AlphaFold3. AutoDockTools 1.5.6 (ADT) was used to perform pre-docking processing of the model, mainly including hydrogen atom addition, charge assignment and atom type assignment. Molecular docking was performed using AutoDock Vina, which is based on Monte Carlo genetic algorithm. Finally, the molecular visualization tool PyMOL 2.6.1 was used to analyze and visualize the docking results.

### Experimental data processing

GraphPad Prism9 software and SPSS 26.0 statistical software were used for data analysis. The differences between the two groups were analyzed by independent sample t test, and non-parametric test was used for comparison between multiple groups. The relationship between OPN, PD-L1 and CD8 expression and clinicopathological features was analyzed by Fisher’s exact probability method, and Spearman correlation analysis was used to compare the correlation between the two molecules. Each experiment in this study was repeated three times independently. *p* < 0.05 was considered statistically significant (*p < 0.05, **p < 0.01, ***p < 0.001, ***p < 0.0001).

## Results

### OPN participates in the anti-tumor immune response mediated by immune cells

**Fig. 1:**
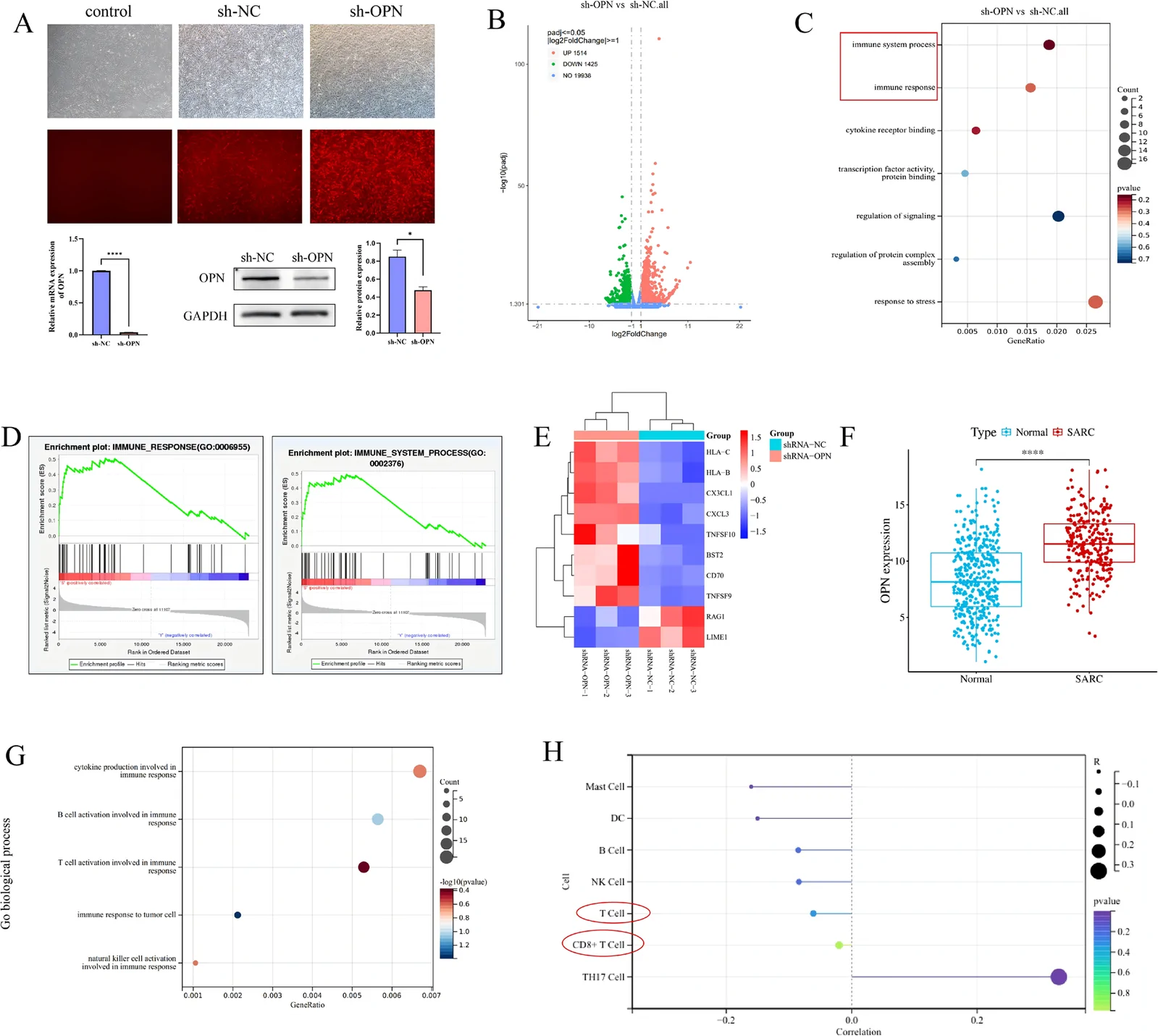
OPN is involved in immune cell-mediated anti-tumor immune response. **A** OPN knockdown lentivirus stable cell line was constructed in SW982 cell line; (**B**) Volcano plot of differentially expressed genes between sh-OPN group and sh-NC group (up-regulated: red; down-regulated: green); (**C**) Bubble plot of differentially enriched genes between sh-OPN group and sh-NC group; (**D**) The differential genes in the sh-OPN group were enriched in the GSEA map of immune response compared with the sh-NC group (“ S “is sh-OPN,” Y “is sh-NC); (**E**) Heatmap of immune-related differential genes between sh-OPN group and sh-NC group; (**F**) Box plot of OPN expression in sarcoma and normal tissues; (**G**) GO analysis of OPN enriched biological functions in sarcomas; (**H**) Lollipop plot of the correlation between OPN and various immune cells in sarcoma

To explore the regulatory mechanism of OPN on SS, we established a stable SW982 cell line with OPN knockeddown using a lentiviral vector. The efficiency of knockdown was verified by qRT-PCR and Western blot which showed reduced OPN expression levels (Fig. 1A). RNA-seq analysis was then performed on OPN knockdown SW982 cells (sh-OPN) and control cells (sh-NC). The results showed that a total of 2939 differentially expressed genes were screened after OPN knockdown compared with sh-NC group, of which 1514 genes were significantly up-regulated and 1425 genes were significantly down-regulated (Fig. 1B). GO functional enrichment analysis revealed thatOPN-associated differentially expressed genes were not only involved in the regulation of proliferation, invasion and migration of SS, but also related to biological processes such as immune system process and immune response (Fig. 1C).Furthermore,, GSEA showed that genes related to immune system regulation and immune response were significantly enriched in the OPN knockdown group (Fig. 1D). Heat map analysis indicated that the expression levels of most immune-related genes were up-regulated in the sh-OPN group (Fig. 1E), suggesting that the inhibition of OPN may stimulate the immune response in SS.

To further explore the regulatory mechanism of OPN on SS immunity, we performed bioinformatics analysis, and gene expression data of sarcoma patients were obtained from TCGA database due to the limitations of SS samples in public databases. The results showed that OPN was also significantly overexpressed in sarcoma tissues compared to normal tissues (Fig. 1F). GO enrichment analysis revealed that OPN related genes were significantly enriched in biological processes including immune response to tumor cells and T cell activation involved in immune response(Fig. 1G). To further explore the relationship between OPN and immunity, we analyzed the correlation between OPN and immune cells. The results showed that OPN expression was negatively correlated with most immune cells, including CD8 + T cells (Fig. 1H).

### OPN induced CD8 + T cell exhaustion participates in immune escape and promotes SS proliferation, invasion and migration

**Fig. 2:**
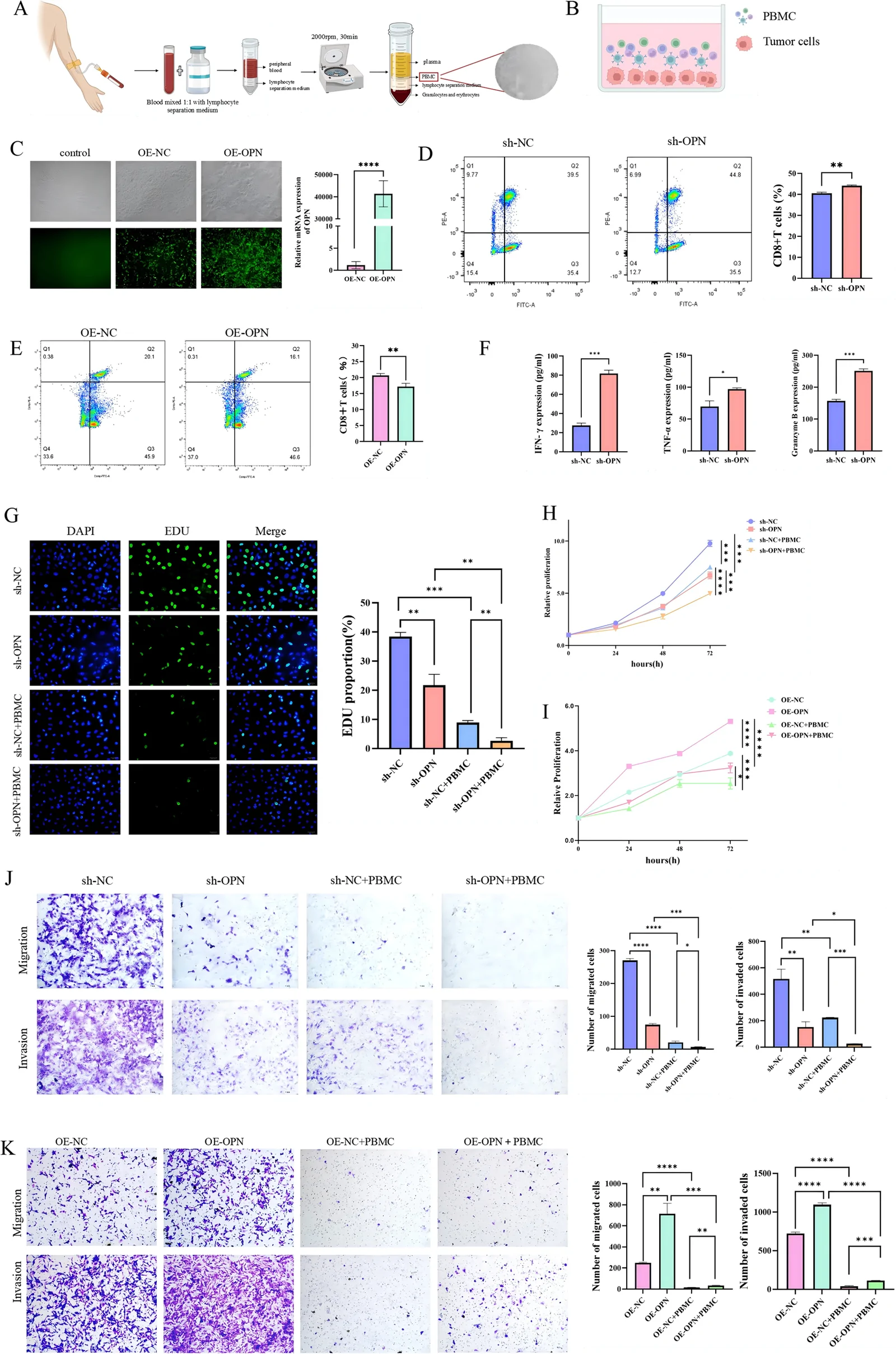
Opn-induced CD8 + T cell immune exhaustion promotes the proliferation, invasion and migration of SS. **A**-**B** Co-culture model of PBMC extracted from peripheral blood and tumor cells; (**C**) OPN overexpression cell line was established in HS-SY-II cells. **D** Flow cytometry was used to detect the percentage of CD8 + T cells after co-culture of PBMC with sh-NC or sh-OPN. **E** Flow cytometry was used to detect the percentage of CD8 + T cells after co-culture of OE-NC and OE-OPN with PBMC. **F** ELISA was used to detect the expression of tumor necrosis factor-α (TNF-α), interferon-γ (IFN-γ) and granzyme B in OPN knockdown group after co-culture; (**G**, **H**, **J**) EDU, CCK8 and Transwell were used to detect the proliferation, invasion and migration ability of SW982 cells co-cultured with PBMC after knocking down OPN. **I**, **K** CCK8 and Transwell were used to detect the proliferation, invasion and migration ability of HS-SY-II cells after overexpression of OPN and co-culture with PBMC

To explore the effect of OPN on CD8 + T cells, we established a co-culture system of SS cells and human PBMC in vitro (SS cells: PBMC = 1:2, culture time for 48 h) to partially simulate the interaction between tumor cells and immune cells in the human tumor immune microenvironment (Fig. 2A-B). A stable cell line overexpressing OPN was constructed using HS-SY-II cells (Fig. 2C). Flow cytometry results showed that in SW982 cells, the percentage of CD8 + T cells in sh-OPN group was significantly higher than that in sh-NC group (Fig. 2D). In contrast, in HS-SY-II cells, the percentage of CD8 + T cells was significantly decreased in the OE-OPN group compared with the OE-NC group (Fig. 2E), indicating that OPN promoted a decrease in the proportion of CD8 + T cells.

Because the function of CD8 + T cells is closely related to the cytokines they secrete, we further measured the levels of IFN-γ, TNF-α, and granzyme B in the co-culture by ELISA (Fig. 2F). The results showed that the expression levels of IFN-γ, TNF-α and granzyme B in sh-OPN group were significantly higher than those in sh-NC group, suggesting that OPN may inhibit the function of CD8 + T cells. Therefore, we preliminarily proposed that OPN may regulate immune response by affecting CD8 + T cells, thereby influencing the malignant biological behavior of SS cells in the tumor immune microenvironment. In SW982 cells, EDU, CCK-8 and Transwell experiments were performed (Fig. 2G, H, J). The comparison between sh-NC and sh-OPN groups showed that OPN knockdown could directly inhibit the proliferation, invasion and migration ability of SS, and our group had previously verified that OPN affected SS invasion and migration through EMT. The results of sh-NC group and sh-NC+PBMC group showed that immune cells inhibited the proliferation, invasion and migration of SS sarcoma. The results of sh-OPN+PBMC group compared with shOPN and sh-NC+PBMC groups showed that under the condition of PBMC co-culture, knockdown of OPN further decreased the proliferation, invasion and migration ability of SS cells. On the contrary, in HS-SY-II cells, CCK-8 and Transwell experiments showed that the comparison between OE-NC and OE-OPN groups showed that the overexpression of OPN could directly promote the proliferation, invasion and migration of SS (Fig. 2I, K). The results of comparison between OE-NC group and OE-NC+PBMC group showed that immune cells inhibited the proliferation, invasion and migration of SS sarcoma. The results of OE-OPN+PBMC group compared with OE-OPN and OE-NC+PBMC groups showed that overexpression of OPN could partially restore the inhibition caused by immune cell attack in OE-NC+PBMC group, and enhance the proliferation, invasion and migration ability of SS cells. This ability was still significantly weaker than that of OE-OPN group.

Taken together, our results suggest that OPN not only directly regulates the signaling pathways related to proliferation, invasion and migration to affect SS, but also participate inimmune escape by inducing CD8 + T cell exhaustion. The specific mechanism require further investigation.

### OPN up-regulates PD-L1 expression in SS cells

**Fig. 3:**
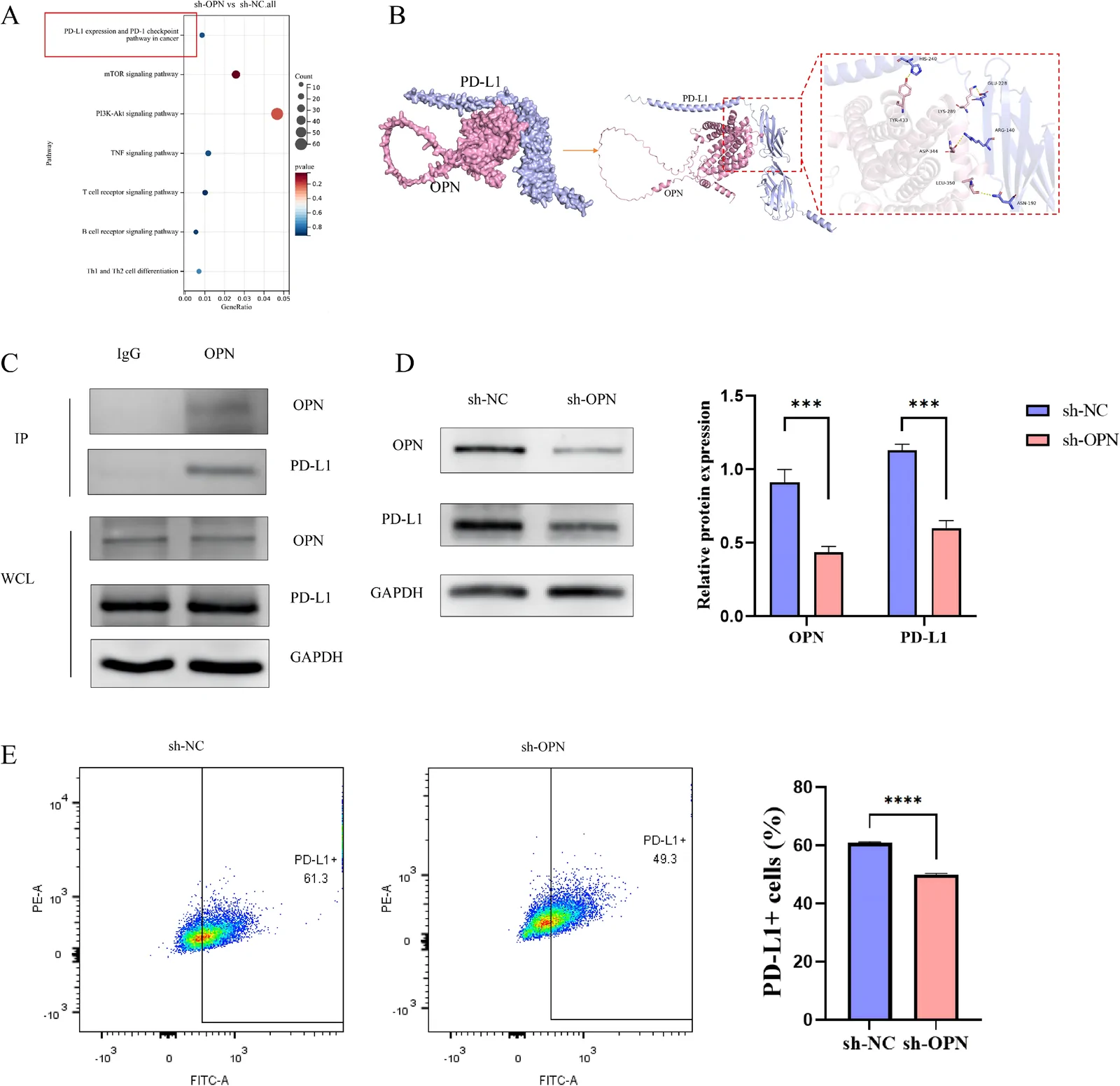
OPN up-regulates PD-L1 expression in SS cells. **A** KEGG map of differentially enriched genes in sh-OPN group compared with sh-NC group; (**B**) molecular docking results of OPN and PD-L1; (**C**) Co-IP was used to verify the interaction between OPN and protein. **D**-**E** WB and flow cytometry were used to detect PD-L1 expression

To further explore how OPN affects the number and function of CD8 + T cells, we performed KEGG enrichment analysis of the differentially expressed genes from the RNA-seq data. Differentially expressed genes were found to be enriched in the PD-L1 expression and PD-1 checkpoint pathway in cancer (Fig. 3A). The binding of PD-L1 to PD-1 on the surface of CD8 + T cells is one of the main mechanisms to induce T-cell exhaustion, and this pathway plays a key role in the immune escape of various tumors. Based on this, we hypothesized that OPN may affect CD8 + T cells by regulating the expression level of PD-L1.

To test this hypothesis, we first predicted the binding possibility between OPN and PD-L1 by molecular docking (Fig. 3B). The binding energy was − 8.4 kcal/mol, indicating a favorable interaction. Subsequently, we verified the protein-protein interaction between OPN and PD-L1 by Co-IP in SW982 cell line (Fig. 3C), indicating that OPN may directly interact with PD-L1 in SS cells. The specific molecular mechanism of this interaction will be explored in future studies. Western blot and flow cytometry were used to detect PD-L1 expression after OPN knockdown. The results showed that the expression level of PD-L1 was significantly decreased when OPN was knocked down (Fig. 3D-E).

### Correlation of OPN, PD-L1 and CD8 expression in SS tissues and their relationship with clinicopathological features

**Fig. 4:**
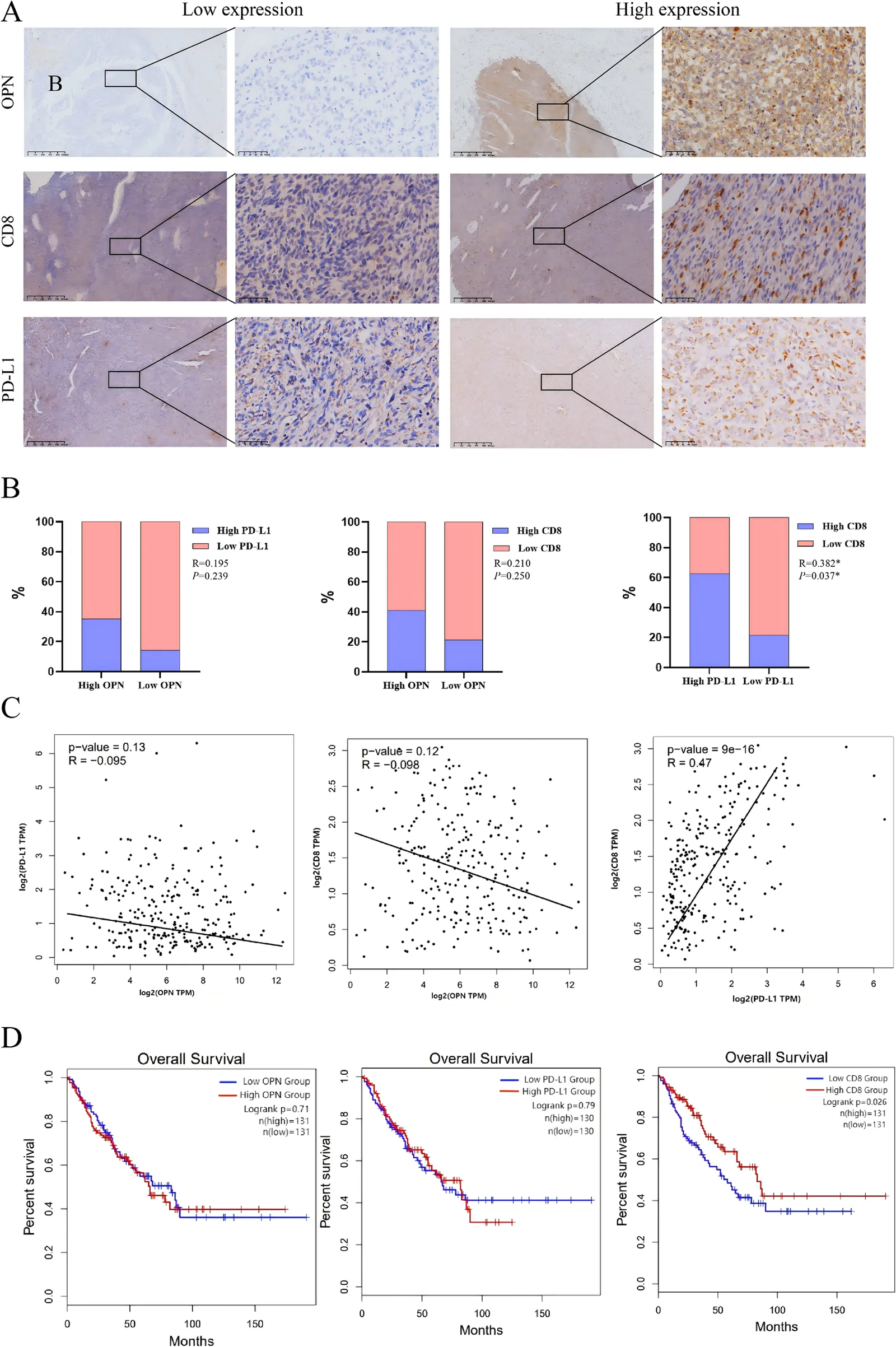
Correlation of OPN, PD-L1 and CD8 expression in SS tissues and its relationship with clinicopathological features. **A** Immunohistochemical staining was used to detect the expression of OPN, PD-L1 and CD8; (**B**) bar stacking plot of correlation between OPN, PD-L1 and CD8 expression; (**C**) Scatter plot of correlation between OPN, PD-L1 and CD8 expression in sarcoma patients in TCGA database; (**D**) Survival analysis of OPN, PD-L1 and CD8 expression levels in sarcoma patients in TCGA database

Immunohistochemical staining was used to detect the protein expression levels of OPN, PD-L1 and CD8 in 31 SS tissue samples. The results showed that OPN was highly expressed in SS tissues with an expression rate of 54.8% (17/31), while PD-L1 and CD8 expression levels were low, and PD-L1 expression rate was 74.2% (23/31). The CD8 expression rate was 67.7% (21/31) (Fig. 4A; Table 1), and the results suggested that SS was a tumor with low T-cell infiltration. Correlation analysis of immunohistochemical results showed that, in samples with low OPN expression, OPN expression exhibited a positive correlation trend with PD-L1 and CD8 expression levels (*p* > 0.05). There was a significant positive correlation between the expression levels of PD-L1 and CD8 (*R* = 0.382, *p* = 0.037) (Fig. 4B). In order to further verify the correlation of OPN, PD-L1 and CD8 mRNA expression levels, we collected OPN, PD-L1 and CD8 expression data in sarcoma tissues through GEPIA2 website and analyzed the correlation. The results showed that OPN was negatively correlated with PD-L1 and CD8, but there was no statistically significant difference (*p* > 0.05). This was consistent with in vitro PCR validation of an increase in PD-L1 expression after knockdown of OPN (Fig. 1 in the Supplementary Material). There was a significant positive correlation between PD-L1 and CD8 (*R* = 0.47, *p* < 0.0001) (Fig. 4C), further supporting the critical role of PD-L1 in regulating CD8 + T cell function.

Further analysis of the relationship between OPN, PD-L1 and CD8 expression levels and the clinicopathological characteristics of SS patients (Table 2) showed no statistically significant associations, which may be attributed to the small sample size (*n* = 31) . Survival analysis could not be performed on these 31 SS patients due to the large time span and high rate of loss to follow-up. To explore the relationship between OPN, PD-L1 ,CD8 expression and survival prognosis, we extracted data of sarcoma patients from the TCGA database and analyzed the relationship between OPN, PD-L1 and CD8 expression levels and survival rate on GEPIA2 website (Fig. 4D). The results showed that the expression levels of OPN and PD-L1 were not significantly correlated with patient survival (*p* > 0.05). Low CD8 expression predicted poor survival (*p* = 0.026).

**Table 2:** The relationship between the expression of OPN, PD-L1, and CD8 in SS tissues and their clinical pathological features

Clinical Feature	n	OPN Expression		p-value	PD-L1 Expression			p-value	CD8 Expression		p-value
		High (n)	Low (n)		High (n)		Low (n)		High (n)	Low (n)	
Sex											
Female	14	9	5	0.473	5	9		0.412	4	10	1
Male	17	8	9		3	14			6	11	
Age (years)											
<40	16	8	8	0.722	4	12		1	3	13	0.135
≥40	15	9	6		4	11			7	8	
Location											
Extremities	18	11	7	0.481	5	13		1	5	13	0.701
Others	13	6	7		3	10			5	8	
Tumor Size (cm)											
<5	7	5	2	0.412	2	5		1	1	6	0.379
≥5	24	12	12		6	18			9	15	
FNCLCC Grade											
I、II	8	2	6	0.097	1	7		0.642	2	6	1
III	23	15	8		7	16			8	15	
TNM Stage											
I、II	8	5	3	0.698	2	6		1	1	7	0.222
III、IV	23	12	11		6	17			9	14	
Metastasis											
Yes	9	6	3	0.456	1	8		0.379	3	6	1
No	22	11	11		7	15			7	15	
Histological Subtype											
BSS	28	15	13	1	6	22		0.156	9	19	1
MFSS	3	2	1		2	1			1	2	

## Discussion

SS is a relatively rare malignant tumor, accounting for 5–10% of all soft tissue sarcomas [8]. It commonly occurs in young adults aged 15–40 years [36] and can occur in any part of the body, among which the limbs are the most common [1, 37]. It has the characteristics of high invasiveness and easy metastasis, and most of the metastasis occurs in the lung, accounting for about 80% [38]. At present, the treatment of SS mainly relies on the combination of surgery, radiotherapy and chemotherapy. The specific treatment plan needs to be determined according to the clinical stage, tumor size, location and general health status of the patient [39, 40]. Relevant clinical studies have shown that about 50% of patients develop local or metastatic recurrence within 5 years [41], and the benefit of adjuvant chemotherapy in local primary SS cases is still controversial [42]. In recent years, immunotherapy has garnered widespread attention as a novel cancer treatment modality due to its remarkable clinical efficacy. Notably, T-cell-based immunotherapies targeting the PD-1/PD-L1 pathway have achieved broad success across various cancers, bringing new hope for the treatment of SS and other soft tissue malignancies. However, immunological studies indicate that SS is generally considered a poorly immunogenic tumor characterized by sparse T-cell infiltration. Livnat et al. used multiple approaches, including scRNA-seq, spatial analysis, and genetic and pharmacological approaches, to explore cancer immune interactions in SS in depth. The results suggest that insufficient immune cell infiltration may be a key mechanism of immune escape in SS [2], which not only provides a new theoretical basis for immune escape in SS, but also provides an important clue to explain why immune checkpoint inhibitors have a low response rate in SS treatment [43–45]. And a recent study by Pollack et al. showed that PD-1/PD-L1 expression and T cell infiltration were lowest in SS among STS [2, 46]. In the present study, immunohistochemical analysis of 31 SS tissues revealed low levels of both PD-L1 expression and CD8 + T-cell infiltration, with a significant positive correlation between the two. Collectively, these findings indicate a prominently immunosuppressive tumor microenvironment (TME) in SS, offering a plausible explanation for the poor clinical efficacy of PD-1/PD-L1 inhibitor monotherapy. Therefore, to improve the overall survival of patients with SS, there is an urgent need to develop novel therapeutic strategies, including but not limited to targeted therapies, immunotherapies, and rationally designed combination regimens.

Our prior LCM and exon array analysis successfully screened 336 DEGs. Notably, ECM-related genes, especially OPN, were significantly enriched in vital SS signaling pathways, indicating their potentially pivotal roles in SS development and progression [20]. OPN, also known as SPP1, EDA-1, BEP-1, and 2ar, is a non-structural ECM protein [47]. It consists of approximately 300 amino acids and is characterized by its highly acidic nature and the presence of O- and N- linked glycosylation [48]. The two primary cell surface receptors for OPN are integrins (such as αvβ3, αvβ5, and αvβ1) and CD44 [49, 50]. OPN is broadly expressed across diverse tissues and organs, predominantly in the bone, breast, kidney, lung, nervous system, pancreas, and skin [51–56]. Furthermore, it is specifically expressed by a variety of immune cell subsets, including T cells, macrophages, B cells, dendritic cells, and NK cells [57–60]. Recently, OPN has increasingly been recognized as a pivotal factor in tumor progression. Accumulating clinical evidence demonstrates that OPN is significantly overexpressed in various malignancies and actively participates in multiple crucial aspects of tumor biology via complex molecular mechanisms [23, 61]. Zhang et al. found that hyperoside regulates OPN by inducing cell cycle arrest, thereby inhibiting the proliferation of osteosarcoma cells and promoting their further differentiation into osteoblasts [62]. Similarly, Jan et al. investigated the regulatory role of OPN in tumor invasion and growth by comparing two glioma cell lines with distinct endogenous OPN secretion levels. Their results demonstrated that inhibiting OPN expression in the highly invasive, OPN-overexpressing U87MG cell line significantly attenuated its invasive capacity [63]. Furthermore, Fan et al. revealed a close correlation between elevated OPN levels and the invasiveness of HCC, observing that various HCC cell lines exhibited enhanced invasive capabilities proportionate to increased OPN expression [64]. In addition, OPN has been found to promote epithelial-mesenchymal transition (EMT), which may be achieved by stabilizing the mesenchymal cytoskeletal protein (vimentin). Silencing vimentin in OPN overexpressing HCC tumor mouse model significantly reduced tumor volume and decreased the expression level of EMT markers [65]. Consistent with these findings, our IHC results showed high OPN expression in SS tissues. Although limited sample sizes precluded statistical significance with some clinicopathological features, OPN clearly plays a critical role in tumor progression and immune regulation. As our previous work proved that OPN drives SS invasion via EMT, we hypothesized that OPN also shapes an immunosuppressive microenvironment to promote immune evasion. RNA-seq of OPN-knockdown SW982 cells revealed through GO analysis that differentially expressed genes were enriched in immune responses. Further GSEA confirmed that OPN knockdown activates immune pathways, demonstrating OPN’s key regulatory role in SS tumor immunity.

OPN was initially identified as an immunomodulatory molecule that activates T cells, known as early T cell activation gene (Eta-1), and regulates T cell activation in malignant tumors [60, 66]. MDSCs and tumor-derived OPN have been shown to disrupt T cell activation and T cell-mediated IFN-γ secretion by interacting with CD44 on T cells, resulting in poor patient survival [67]. Jun Wei et al. found an increased frequency of T cells producing IFN-γ, TNF-α, and IL-12 in spleen, blood, and/or brain samples from OPN-deficient mice, suggesting that OPN inhibits effector T cell activity [31]. Furthermore, Zhang et al. [34] demonstrated that CD4 + T-cell activity was significantly diminished during co-culture of macrophages with cancer cell lines; notably, inhibiting OPN expression restored this activity. This finding suggests a critical role for OPN in mediating macrophage-induced suppression of T cells. Consistent with these mechanisms, our GO enrichment analysis revealed that OPN-associated genes in sarcoma tissues were significantly enriched in biological processes related to tumor cell immune responses and T-lymphocyte activation. Meanwhile, OPN expression was negatively correlated with the infiltration of most immune cells, particularly CD8 + T cells. These results collectively indicate that OPN not only participates in regulating the immune response within the tumor microenvironment but also likely facilitates tumor immune evasion by dampening immune cell activity. Scott R Walsh et al. showed that in cellular immunotherapy, it is usually through enhancing the activity of killer T cells, that is, CD8 + T cells, to achieve a stronger anti-tumor effect [68]. Typically, CD8 + T cell exhaustion is marked by a decrease in cytokine production [69]. Herein, by co-culturing PBMCs with two SS cell lines subjected to OPN knockdown or overexpression, we demonstrated that OPN likely regulates immune responses by inducing the quantitative and functional exhaustion of CD8 + T cells. These findings further substantiate the potential role of OPN in SS immune evasion. Tumors suppress host immunity via various mechanisms, notably by upregulating surface PD-L1. The subsequent interaction between PD-L1 and PD-1 on antigen-specific CD8 + T cells induces severe functional decline, serving as the principal driver of CD8 + T cell exhaustion [70, 71]. In HCC, OPN promotes PD-L1 expression by activating the CSF1-CSF1R pathway in macrophages, and blockade of PD-L1 and CSF1R leads to increased CD8 + T cell infiltration [33]. To date, the specific regulatory mechanism of OPN on PD-L1 in SS has not been reported. In this study, RNA-seq analysis revealed that differentially DEGs were significantly enriched in the PD-1/PD-L1 pathway. Molecular docking and co-IP assays indicated that OPN may regulate this pathway by directly binding to PD-L1, representing the first proposal of a potential direct interaction between OPN and PD-L1. In vitro experiments demonstrated that OPN knockdown led to a decrease in PD-L1 protein expression, consistent with our IHC findings of a positive correlation between OPN and PD-L1 at the protein level. Paradoxically, analysis of public mRNA datasets revealed an inverse correlation between OPN and PD-L1 at the transcriptional level, a trend further validated by our qPCR results. We hypothesize that this discrepancy may stem from post-transcriptional regulation, epigenetic modifications (such as methylation), or other mechanisms affecting mRNA-to-protein translation efficiency, which will be further elucidated in our future research.

In summary, we preliminarly suggest that OPN induces CD8 + T cell immune exhaustion by regulating PD-L1 expression, thereby promoting immune escape in SS. This finding not only reveals the key role of OPN-PD-L1/CD8 + T cell axis in SS immune escape, but also suggests that OPN may become a new target for SS immunotherapy, which provides a theoretical basis for the development of new immunotherapy strategies. However, our study has certain limitations. First, the lack of flow cytometric analysis for classic T cell exhaustion markers (e.g., PD-1, TIM-3, and LAG-3) precludes the definitive confirmation of CD8 + T cell exhaustion. Second, inter-observer agreement was not evaluated for the IHC scoring. Third, regarding the external validation using the TCGA database, the absence of histological subtype annotations for SS hindered our ability to evaluate the correlations among OPN, PD-L1, and CD8 across distinct SS subtypes. Most importantly, the specific mechanisms underlying OPN-mediated PD-L1 upregulation and subsequent CD8 + T cell functional exhaustion remain to be further validated in in vivo animal models. In the future, we will further extend and verify the findings of this study, and clarify the direct regulatory mechanism of OPN on PD-L1 in SS in detail, so as to provide a more solid theoretical basis for the clinical application of OPN as a new target for SS immunotherapy.

## Supplementary Information

Supplementary Material 1.

Supplementary Material 2.

## Data Availability

The custom code used for data processing, analysis, and figure generation in this study has been deposited in a public repository and is accessible via the following link: https://doi.org/10.6084/m9.figshare.32592273 .
