# Supplementary material for: Preliminary study on the mechanism of OPN up-regulating PD-L1 to induce CD8 + T cell exhaustion in synovial sarcoma
Source: BMC Cancer. 2026 Jul 3;26:1172. doi: 10.1186/s12885-026-16363-x (PMC13632446; doi:10.1186/s12885-026-16363-x)
Supplement: Supplementary file 1 [file 12885_2026_16363_MOESM2_ESM.pdf]

Supplementary original figures of Western blotting

We detected and confirmed the molecular weights of the target antigens: OPN at 90 kDa and PD-L1 at 47 kDa. In the article show images we marked with red boxes.

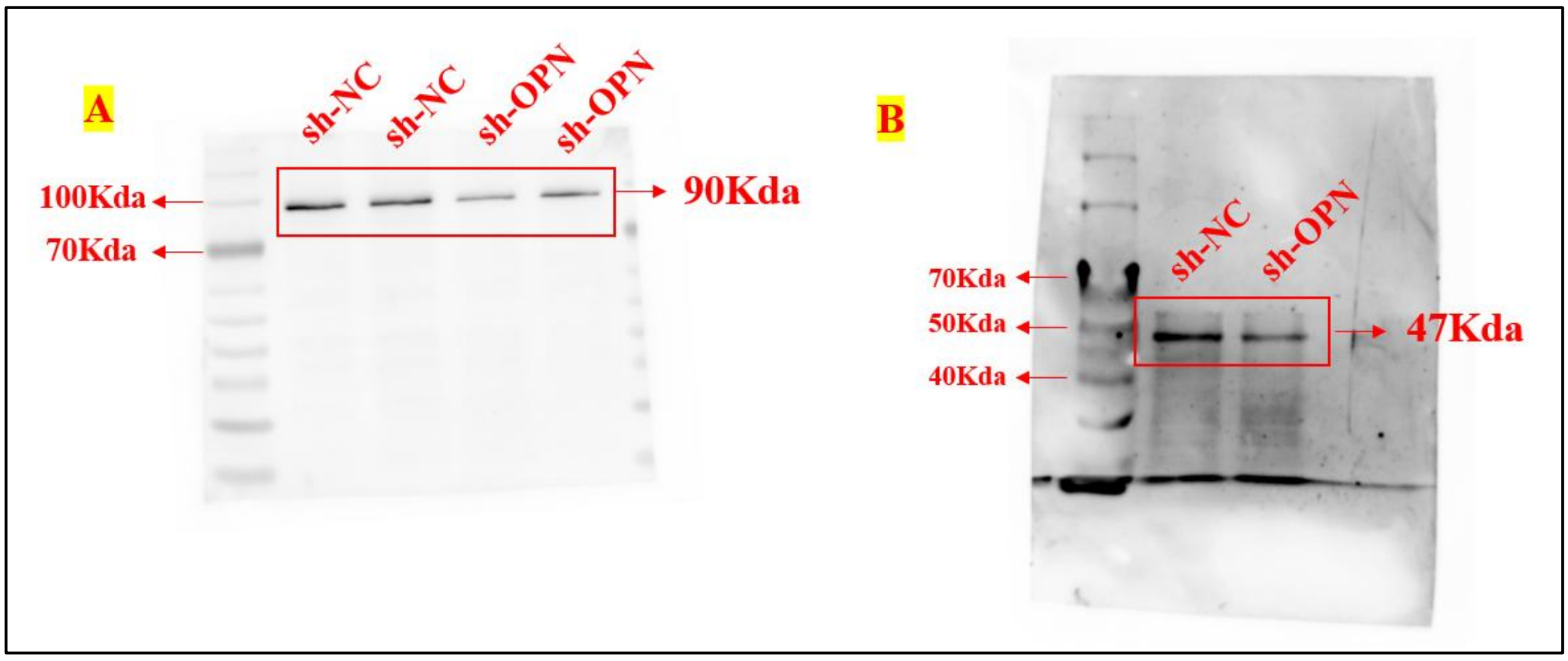

Figure 1 (A) shows the full-length, original, and unprocessed Western blot image of the anti-OPN antibody; (B) shows the full-length, original, and unprocessed Western blot image of the anti-PD-L1 antibody, used to confirm the specific detection results of the target antigens.

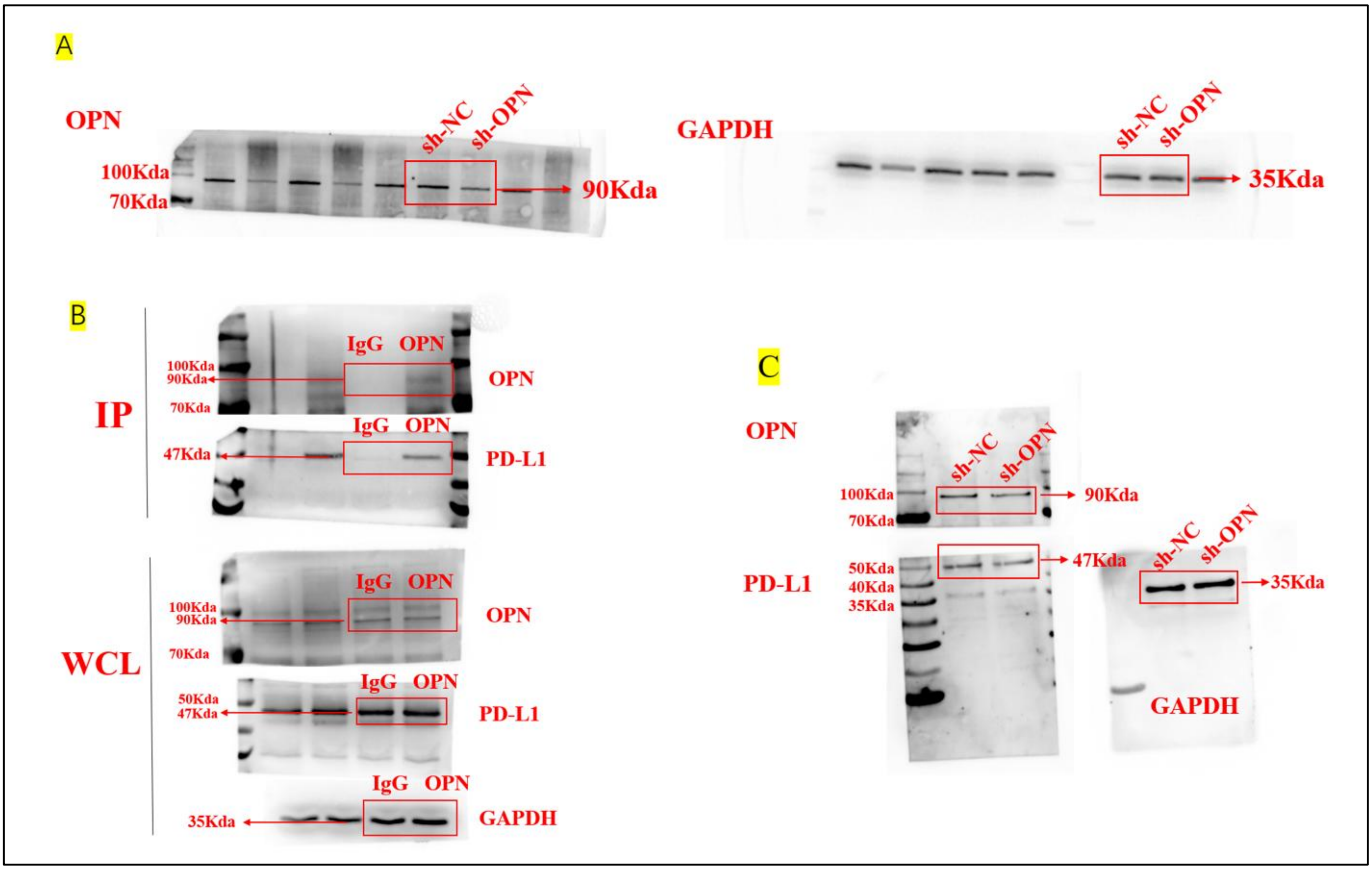

Figure 2 Western Blot assay of Figure 1A, Figure 3C, and Figure 3D.

(A) The original Western blot images of Figure 1A. (B) The original Western blot images of Figure 3C. (C) The original Western blot images of Figure 3D.

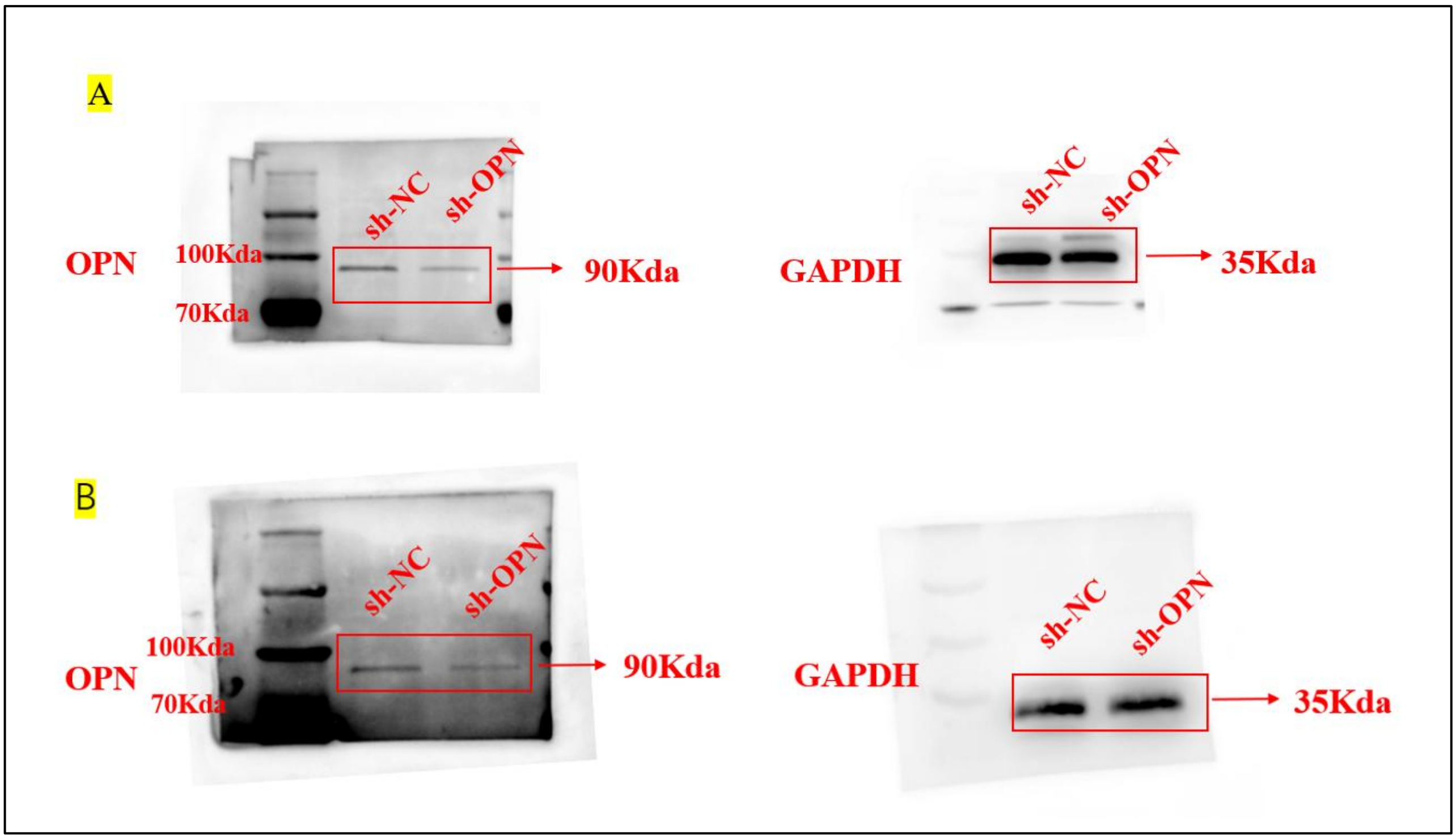

Figure 3 Replicate Western blot assay of OPN knockdown cells.

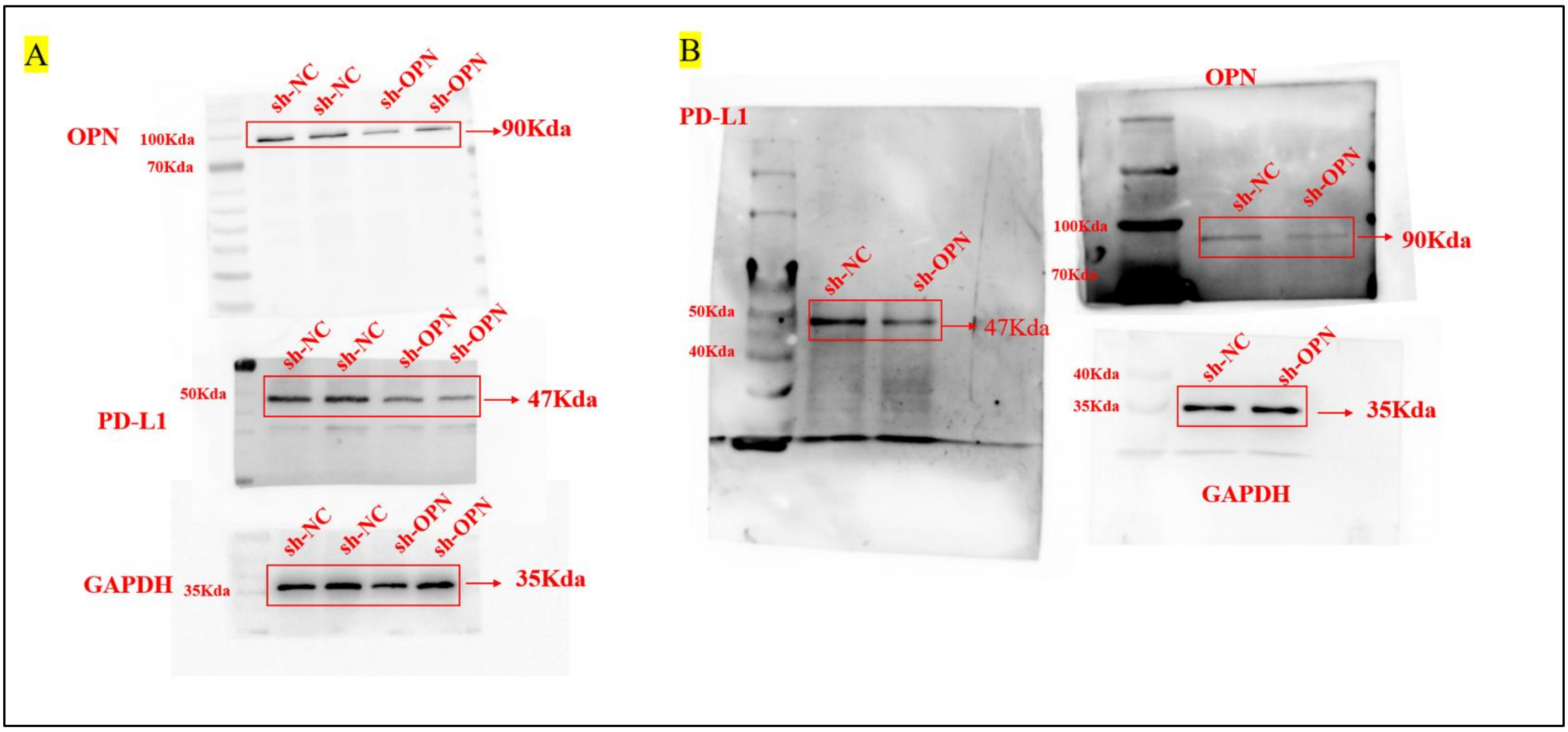

Figure 4 Replicate Western blot assay of PD-L1 protein expression after OPN knockdown in cells.
