# Supplementary material for: Preliminary study on the mechanism of OPN up-regulating PD-L1 to induce CD8 + T cell exhaustion in synovial sarcoma
Source: BMC Cancer. 2026 Jul 3;26:1172. doi: 10.1186/s12885-026-16363-x (PMC13632446; doi:10.1186/s12885-026-16363-x)
Supplement: Supplementary file 2 [file 12885_2026_16363_MOESM1_ESM.docx]

Table 1 Clinicopathological information of 31 patients with SS

| ID | Gender | Age (years) | Location | Tumor Diameter (cm) | FNCLCC Grade | TNM Stage | Diagnosis | Metastasis |
| --- | --- | --- | --- | --- | --- | --- | --- | --- |
| S1 | Female | 40 | Lower left femur | 7.5 | Grade III | Stage IIIA | BSS | No |
| S2 | Male | 36 | Left thigh | 5 | Grade III | Stage IIIA | BSS | No |
| S3 | Female | 40 | Right interthigh | 2.8 | Grade III | Stage II | MFSS | No |
| S4 | Male | 55 | Left iliac region | 10.3 | Grade III | Stage IV | BSS | Yes |
| S5 | Female | 22 | Left heel | 4.5 | Grade III | Stage II | BSS | No |
| S6 | Male | 36 | Right elbow joint | 11 | Grade III | Stage IIIB | BSS | No |
| S7 | Male | 19 | Left lower leg, ankle | 8.5 | Grade III | Stage IIIA | BSS | No |
| S8 | Female | 14 | Right elbow with bone marrow metastasis | 10.5 | Grade III | Stage IV | BSS | Yes |
| S9 | Female | 15 | Right neck | 4 | Grade III | Stage II | BSS | No |
| S10 | Female | 32 | Left groin | 4.5 | Grade III | Stage II | BSS | No |
| S11 | Female | 38 | Left forearm | 9 | Grade II | Stage IIIA | BSS | No |
| S12 | Male | 32 | Right lower posterior molar region | 3 | Grade III | Stage II | BSS | Yes |
| S13 | Female | 37 | Left ischial bursa / pubis | 7.5 | Grade III | Stage IV | BSS | No |
| S14 | Male | 70 | Left hip | >5 | Grade III | Stage IV | BSS | Yes |
| S15 | Female | 22 | Left femoral lesser trochanter | 2.5 | Grade III | Stage II | BSS | No |
| S16 | Female | 21 | Right thigh mass | 7.5 | Grade III | Stage IIIA | BSS | No |
| S17 | Male | 55 | Right foot mass | 5.2 | Grade III | Stage IV | BSS | Yes |
| S18 | Male | 47 | Left anterior tibia | 5 | Grade III | Stage IV | MFSS | Yes |
| S19 | Male | 39 | Left hand and forearm | 6 | Grade III | Stage IIIA | BSS | Yes |
| S20 | Female | 55 | Left thigh | 6 | Grade III | Stage IV | BSS | No |
| S21 | Male | 56 | Left thigh mass | 8 | Grade III | Stage IIIA | BSS | No |
| S22 | Male | 52 | Right kidney | 5 | Grade III | Stage II | BSS | No |
| S23 | Female | 61 | Gallbladder | 10 | Grade II | Stage IIIA | BSS | No |
| S24 | Female | 54 | Right lung | 16 | Grade II | Stage IIIB | BSS | No |
| S25 | Male | 51 | Left upper limb mass | 10 | Grade II | Stage IIIA | BSS | No |
| S26 | Male | 49 | Neck | 19 | Grade II | Stage IIIB | MFSS | No |
| S27 | Male | 24 | Right upper lobe of lung | 6 | Grade III | Stage IV | BSS | Yes |
| S28 | Female | 35 | Neck | 1.2 | Grade II | Stage II | BSS | No |
| S29 | Male | 17 | Right upper limb | 7 | Grade II | Stage IIIA | BSS | No |
| S30 | Male | 47 | Right lower limb | 5.5 | Grade II | Stage IIIA | BSS | No |
| S31 | Male | 58 | Popliteal fossa cyst | 6.5 | Grade III | Stage IIIA | BSS | Yes |

Figure 1. PCR analysis of PD-L1 expression after OPN knockdown.
